# Towards real-world neuroscience using mobile EEG and augmented reality

**DOI:** 10.1038/s41598-022-06296-3

**Published:** 2022-02-10

**Authors:** Alexandra Krugliak, Alex Clarke

**Affiliations:** Department of Psychology, University of Cambridge, Cambridge, UK

**Keywords:** Neuroscience, Cognitive neuroscience, Computational neuroscience, Visual system

## Abstract

Our visual environment impacts multiple aspects of cognition including perception, attention and memory, yet most studies traditionally remove or control the external environment. As a result, we have a limited understanding of neurocognitive processes beyond the controlled lab environment. Here, we aim to study neural processes in real-world environments, while also maintaining a degree of control over perception. To achieve this, we combined mobile EEG (mEEG) and augmented reality (AR), which allows us to place virtual objects into the real world. We validated this AR and mEEG approach using a well-characterised cognitive response—the face inversion effect. Participants viewed upright and inverted faces in three EEG tasks (1) a lab-based computer task, (2) walking through an indoor environment while seeing face photographs, and (3) walking through an indoor environment while seeing virtual faces. We find greater low frequency EEG activity for inverted compared to upright faces in all experimental tasks, demonstrating that cognitively relevant signals can be extracted from mEEG and AR paradigms. This was established in both an epoch-based analysis aligned to face events, and a GLM-based approach that incorporates continuous EEG signals and face perception states. Together, this research helps pave the way to exploring neurocognitive processes in real-world environments while maintaining experimental control using AR.

## Introduction

A great deal of progress has been made in cognitive neuroscience, where imaging techniques offer a window into the mysteries of visual perception, memory, attention and language, to name a few. Such success has largely been achieved with a scientific approach where researchers seek to isolate specific cognitive functions and study their neurocognitive instantiation in a controlled manner. This is an important and fruitful approach, and will continue to be so. As cognitive neuroscience methods progress, a complementary approach has also become more feasible, namely, research using more naturalistic paradigms which look towards the functioning of the human mind unleashed from controlled experiments^1–4^. Moreover, experimentation beyond the lab is possible by building on the platform established by prior approaches, and by employing newer and emerging technologies to study the human brain—such as virtual reality, augmented reality and fully mobile approaches for neural recordings. Going further, combining these approaches could offer a toolkit that allows for the study of neural function in uncontrolled complex visual environments, getting us closer to studying cognition in our natural habitat. Here, we present and validate an approach to studying human cognition in naturalistic, real-world environments, while importantly retaining the ability to manipulate our key variables and retain experimental control. We achieve this by combining mobile EEG (mEEG) with head-mounted cameras and augmented reality (AR).

Mobile whole-head EEG applications have made great strides in the past decade, recently being applied to study memory^5–7^, emotion^8,9^, attention^10,11^ and movement^12–14^ in complex real-world settings. Advances in hardware and software have made fully mobile high-density EEG a useful tool for cognitive neuroscience^15,16^, however, what remains problematic is the ability to flexibly manipulate key variables, as now our cognitive variables of interest are part of the real world. For some disciplines, this could be circumvented by placing certain objects in certain places, thereby constructing experimentally useful environments (e.g.^6^). However, a more adaptable and flexible approach is to utilise immersive head-mounted displays to present virtual objects on the background of the real world, allowing for full experimental control over what people see and where those items are located. In contrast to virtual reality, where the whole environment is simulated, AR affords the ability to place 3D virtual objects in the actual environment. Recent research suggests that AR is more engaging than VR, with an indication of improved memory performance^17^. The ability to place a limitless set of virtual items in the real world offers a degree of experimental control that can’t be matched through brute force methods. Here, we propose that by combining mobile EEG with head-mounted AR, we can study human cognition and neuroscience in real-world settings whilst also manipulating the world people see.

In order to demonstrate the feasibility of such an approach, we chose to look at the face inversion effect^18^. Typically, inverted faces produce a stronger response in low frequency power compared to upright faces. This effect is suited for our purposes as it is well defined and robust, and is therefore more likely to be observable under potentially unfavourable conditions—such as participants walking around freely while viewing virtual, but not perfectly realistic, stimuli. In our experiment, participants completed three face inversion tasks while we recorded 64-channel EEG with a mobile system. The tasks increased in technical complexity, aiming to validate the use of mobile EEG under free moving conditions in combination with virtual 3D objects presented through a head-mounted AR device. First, a computer-based face-inversion task served as a control condition. Second, participants viewed photographs of real faces attached to the walls of a corridor. Finally, participants viewed virtual faces through the head-mounted AR device. In both the second and third task, participants were freely navigating in an indoor corridor setting, and we could contrast EEG activity for upright and inverted faces. As a follow on, we looked to establish routines whereby we could relate the dynamically unfolding visual environment to the dynamic neural signatures recorded while participants walked through natural environments. This is important, as to be able to study cognition in real-world settings, we need methods to link natural dynamic behaviour to dynamic neural signals. To achieve this, we used a GLM approach similar in nature to that used in naturalistic fMRI studies of movie watching (e.g.^19,20^) and MEG studies of language comprehension (e.g.^21^), again testing the sensitivity of the approach against face inversion effects. Together, these twin analyses demonstrate an approach that manipulates and controls variables in conjunction with mobile neural recordings to reveal cognitive effects in dynamically changing settings.

## Methods

### Participants

**Figure 1:**
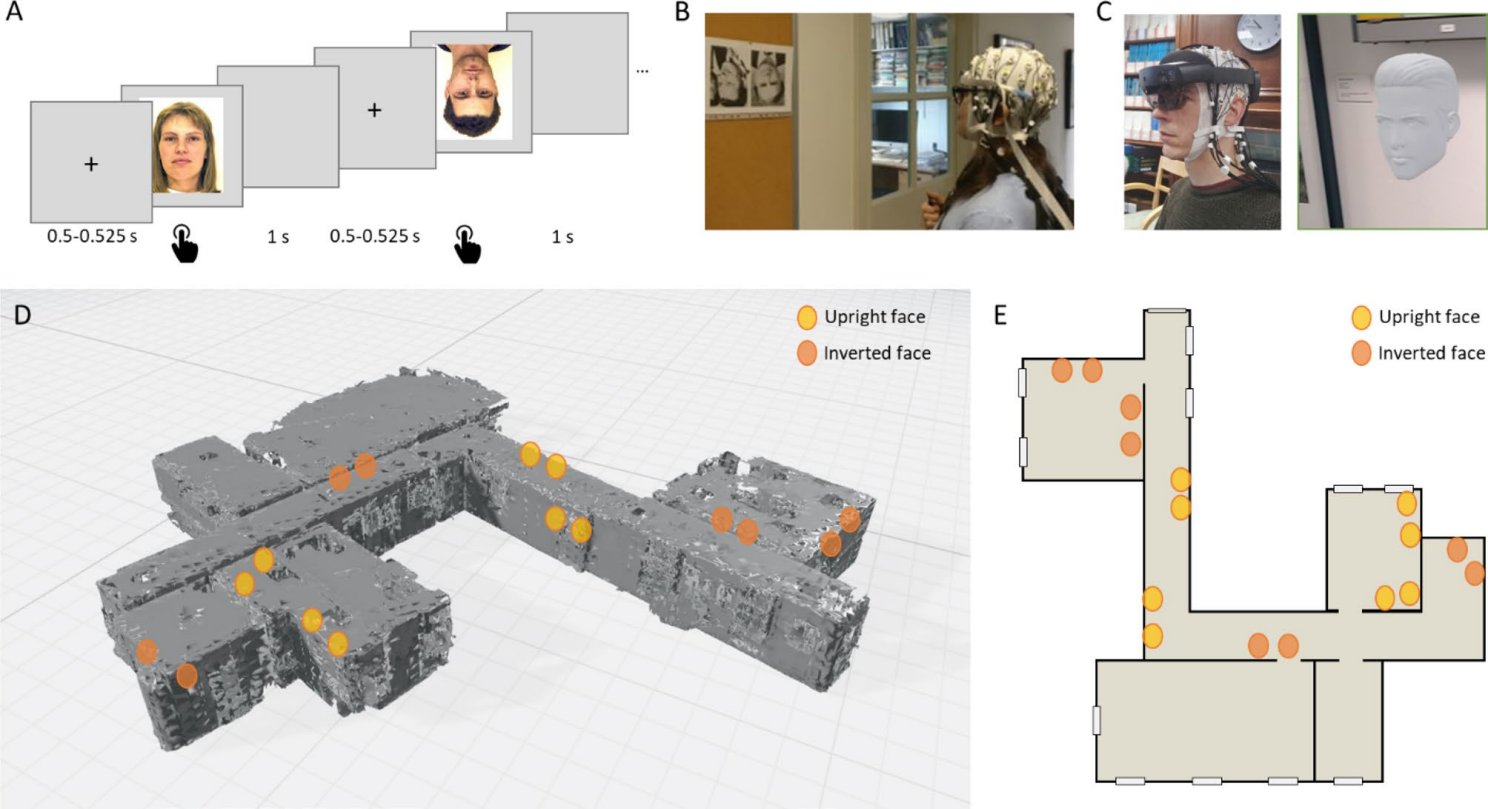
Experimental setup. (**A**) Computer-based task showing example trials and timings. (**B**) Mobile EEG setup and face photos. (**C**) Mobile EEG and AR setup and example virtual face used for AR task. The photographs in B and C show the authors, used with permission. (**D**) 3D spatial map created by the Hololens 2 of the experimental environment, showing approximate locations of upright (yellow) and inverted (orange) virtual faces. (**E**) 2D map of environment showing approximate locations of upright (yellow) and inverted (orange) virtual faces.

Eight healthy participants (age 19–38 years, 4 females) took part in the study, and all had normal or corrected to normal vision. The experiment was conducted in accordance with the declaration of Helsinki and approved by the Cambridge Psychology Research Ethics Committee. All participants gave informed consent. Additionally, two participants who are the authors, A.K. and A.C., gave informed consent to publish their photographs in Fig. 1B and C respectively.

### Stimuli and procedure

The experiment had three distinct tasks.

Task 1, Computer-based: A computer-based face processing task was conducted using upright and inverted images of a male and female face (Fig. 1A). The two face images were obtained from the Psychological Image Collection at Stirling (pics.stir.ac.uk). Face images were presented in colour, were front facing on a white background and had a neutral expression. Each face was shown in both upright and inverted orientations. Participants were instructed to press a button on the keyboard when they had seen the face. Each trial began with a fixation cross lasting between 500 and 525 ms, followed by the face image lasting until the button press, before a blank screen lasting 1 s. A total of 200 trials were presented, 100 upright and 100 inverted faces. The experiment was presented using Psychtoolbox version 3 and Matlab R2019b, and triggers recorded via a USB to TTL module (https://www.blackboxtoolkit.com).

Task 2, mEEG + photos: For task 2, photos of upright or inverted faces were attached to the walls of the corridor and participants were asked to view the faces while walking along the corridor (Fig. 1B). Sixteen black and white photographs of faces (8 upright, 8 inverted) were taken from the set used by Greene and Hodges^22^. Faces were forward facing with natural expressions and plain backgrounds. In addition to EEG, participants were fitted with a head-mounted camera attached to a Raspberry Pi Zero (https://www.raspberrypi.org/). Participants were asked to move along the corridor, and into side rooms*,* and pause at each face image. When they were viewing the image they pushed a button which sent a signal to the LiveAmp trigger input. The condition the image belonged to could then be derived from the head-mounted video. Participants repeatedly viewed the face images resulting in an average of 42 upright (range 24–70) and 39 inverted trials (range 22–72). The variable number of trials across participants reflects the different routes participants chose to take.

Task 3, mEEG + AR: In task 3, a Microsoft Hololens 2 AR device (https://www.microsoft.com/en-us/hololens) was positioned over the electrodes (Fig. 1C). The clear lenses of the Hololens allow the participant to see the actual environment, while virtual faces were presented anchored to specific locations in the corridor (Fig. 1D,E). The onboard camera of the Hololens captured first-person video, including the virtual faces. The virtual faces were obtained from the Microsoft 3D objects library, and were white heads with texture and hair, but no colour (Fig. 1C). In this respect, they resembled white marble heads without a body. Four different virtual heads were used (2 male, 2 female), with each head appearing in 4 locations, half of which the faces were inverted. This resulted in a total of 16 heads, 8 of which were upright and 8 inverted. The virtual heads were placed along the same corridor and side rooms as used for the face photographs (which were not present during task 3). Participants paused at each virtual face and pushed a button, and saw each face numerous times resulting in an average of 63 upright (range 28–90) and 53 inverted trials (range 27–87).

### EEG recording

EEG was recorded using the Brainvision LiveAmp 64 mobile system (Brain Products GmbH, Gilching, Germany). In all tasks we recorded 64-channel EEG through ActiCap Slim active Ag/AgCl electrodes referenced to an electrode placed at FCz, with a sampling rate of 500 Hz. Electrodes were embedded in an elastic cap with electrode locations conforming to the international 10/20 system. Electrode cables were carefully routed through the cable ties and kept flat to the head to minimise cable sway during recording. EEG signals were amplified using two wireless amplifiers and recorded to onboard memory cards. Data recording was controlled using BrainVision Analyzer, and during setup, impedances were reduced to below approximately 5–10 kOhm.

### Epoch-based analysis

**Figure 2:**
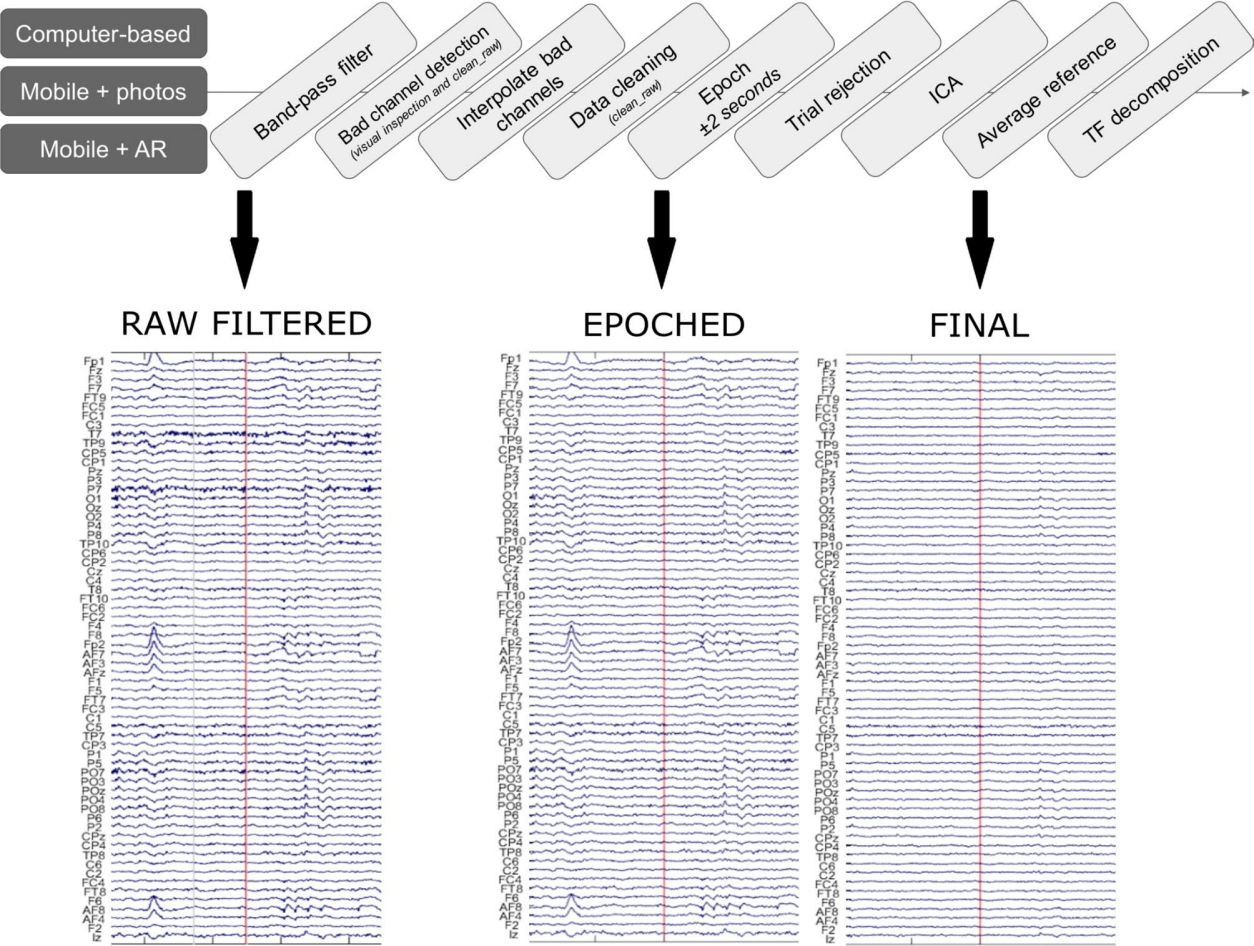
EEG preprocessing. Core methods across task, and example data at different stages of processing from the Mobile + AR task.

The same EEG analysis pipeline was independently used for the data from all three tasks, with a slight modification for the mobile tasks (2 and 3; Fig. 2). Raw EEG signals were imported into EEGlab^23^ and cropped to just before the first trial and just after the last trial, before channel locations and names were imported. The data were then band-pass filtered using a onepass zerophase Blackman-windowed sinc FIR filter with transition width 0.1 Hz and order 27500. Band-pass were 0.5 to 40 Hz for task 1, and 1 to 20 Hz for task 2 and 3. The narrower filter range for task 2 and 3 was due to the additional noise suppression this afforded, and the frequency range of interest identified in task 1. Bad channels were detected based on automated procedures (pop_rejchan.m), and a channel was classified as bad based on probability (threshold 3 SDs), spectrum (threshold 3 SDs) and kurtosis measures (threshold 5 SDs). Any identified bad channels were subsequently interpolated using spherical interpolation (task 1: mean = 9.4, range = 7–14, task 2: mean = 5.9, range = 1–11; task 3: mean = 7.8, range = 4–12). The continuous data was then cleaned using clean_artifacts. For task 1, the data were epoched between − 1 and 2 s around the onset of the face images, and for task 2–3 the data were epoched between − 2 and + 2 s centred on the button press. The condition of each epoch was then determined using the head-mounted videos. The epoched data was visually inspected and noisy trials were removed. Noisy trials were based on visual inspection of the data, specifically trials that contained high frequency noise or large amplitude signals beyond the range of normal activity (task 1: median = 5%, mean = 10%, range = 0–47%; task 2: median = 8%, mean = 7%, range = 0–14%; task 3: median = 7%, mean = 9%, range = 1–19%).

We used ICA to focus our analysis on signals coming from the brain rather than external or noise sources. ICA was applied to the epoched data using runica with the extended and pca options, and extracted N components where N was 64 minus the number of bad channels. ICs were analysed using ICLabel^24^ to identify components related to brain activity to retain when reconstructing the EEG data. Components were retained if they had a greater than 20% chance of reflecting brain activity but could not have more than 20% chance of reflecting any artefact classification (eyes, cardio, muscle, channel noise). These components were then visually inspected and any remaining components not showing a 1/f characteristic (i.e. more power at low frequencies compared to higher frequencies) or with only frontal electrode weights were additionally removed. On average 13.9 components were retained in task 1 (range 5–30), 6.4 in task 2 (range 2–11) and 8.3 in task 3 (range 4–13). We used this very selective approach to help focus the EEG signals on cognitively relevant signals. After ICA, the data were again visually inspected before transforming the signals using an average reference. The processed EEG signals were converted to Fieldtrip^25^, and time-frequency representations of each trial were calculated using Morlet wavelets between 4 and 35 Hz in 25 ms time steps between − 0.25 and 1.25 s (task 1), or 4–20 Hz in 25 ms time steps between − 1.5 and 1.5 s (task 2 and 3). No baseline correction was applied.

We contrasted low frequency oscillatory power averaged between 5 and 15 Hz based on prior studies showing enhanced power for inverted compared to upright faces in this range. Further, as these effects are expected over posterior electrodes, we initially focused our analysis on these electrodes. To do this, power was averaged across posterior electrodes (N = 17, defined as all occipital, parietal and parietal-occipital electrodes) and between 0 to 400 ms (task 1) and − 1.5 to 1.5 s (task 2 and 3). This produced one value per trial before the data were converted to a standardized z-score. A linear mixed effects model was used to model trial-wise power with a predictor variable specifying if the trial was an upright face (− 0.5) or an inverted face (0.5), and including a random effect of Subject (EEG_power ~ condition + (1|Subject)). Trials with a value more than 4 standard deviations away from the mean were additionally removed prior to the mixed effects model (task 1: 0.5% of data; task 2: 0.2%; task 3: 0.2%).

### GLM-based analysis

The GLM-based approach was applied to the mobile tasks (2 and 3). We took the ICs selected for the epoch-based analysis, and applied them to the filtered data from each task. We then created an additional stimulus channel in the data, by inserting a stick function at the time of each face event with values rising to 0.5 for an inverted face and − 0.5 for an upright face. All other time points were set to zero. This stimulus channel was then convolved with a 2 s hamming window centred on the face events. The continuous EEG was then visually inspected, and noisy time segments were selected and cropped from the data (task 2: mean = 1.3%, range 0.1–4.7%; task 3: mean = 7.4%, range = 0–17.3%).

The EEG was imported into Fieldtrip^25^, and a time-frequency representation of the data were calculated using Morlet wavelets between 4 and 20 Hz in 25 ms time steps. No baseline correction was applied, and time-frequency signals were averaged across 5–15 Hz (following the epoch-based analysis). This resulted in a low frequency power time-series at each electrode. To test for face inversion effects over posterior electrodes, a general linear model was run fitting the power averaged over posterior electrodes with predictors of the face inversion channel, and three additional predictors of no-interest defined by the three accelerometer channels (amplifier motion in x, y and z directions) using fitglm in Matlab. Prior to the GLM, data were converted to a standardized z-score.

### Participant motion and EEG

Finally, we quantified how participant motion, measured by the accelerometers within the amplifiers which were attached to the participants back, related to EEG signal amplitudes. To do this, the continuous data (EEG channels plus 3 accelerometer channels) were split into 2 s non-overlapping chunks, and the root mean square (RMS) calculated for each channel. The RMS was averaged across the three accelerometer channels, creating one value per 2 s period. These values were then binned into low, medium and high motion RMS groups using the discretize function in Matlab, before averaging the EEG RMS according to the motion RMS bins (Task 2: Low motion RMS bin edges 1.6–10.5, mean number of segments = 15.9; Medium motion RMS bin edges 10.5–19.4, mean number of segments = 97.5; High motion RMS bin edges 19.4–28.3, mean number of segments = 27.8; Task 3: Low motion RMS bin edges 0.5–13.4, mean number of segments = 25.8; Medium motion RMS bin edges 13.4–26.3, mean number of segments = 189.1; High motion RMS bin edges 26.3–39.1, mean number of segments = 39.3). This resulted in an EEG RMS value for each electrode and each of the low, medium and high RMS bins. A linear mixed effects model was used to test if the EEG RMS values related to the levels of participant motion (as defined by the motion RMS bins).

## Results

### Epoch-based analysis

#### Task 1: computer face inversion task

**Figure 3:**
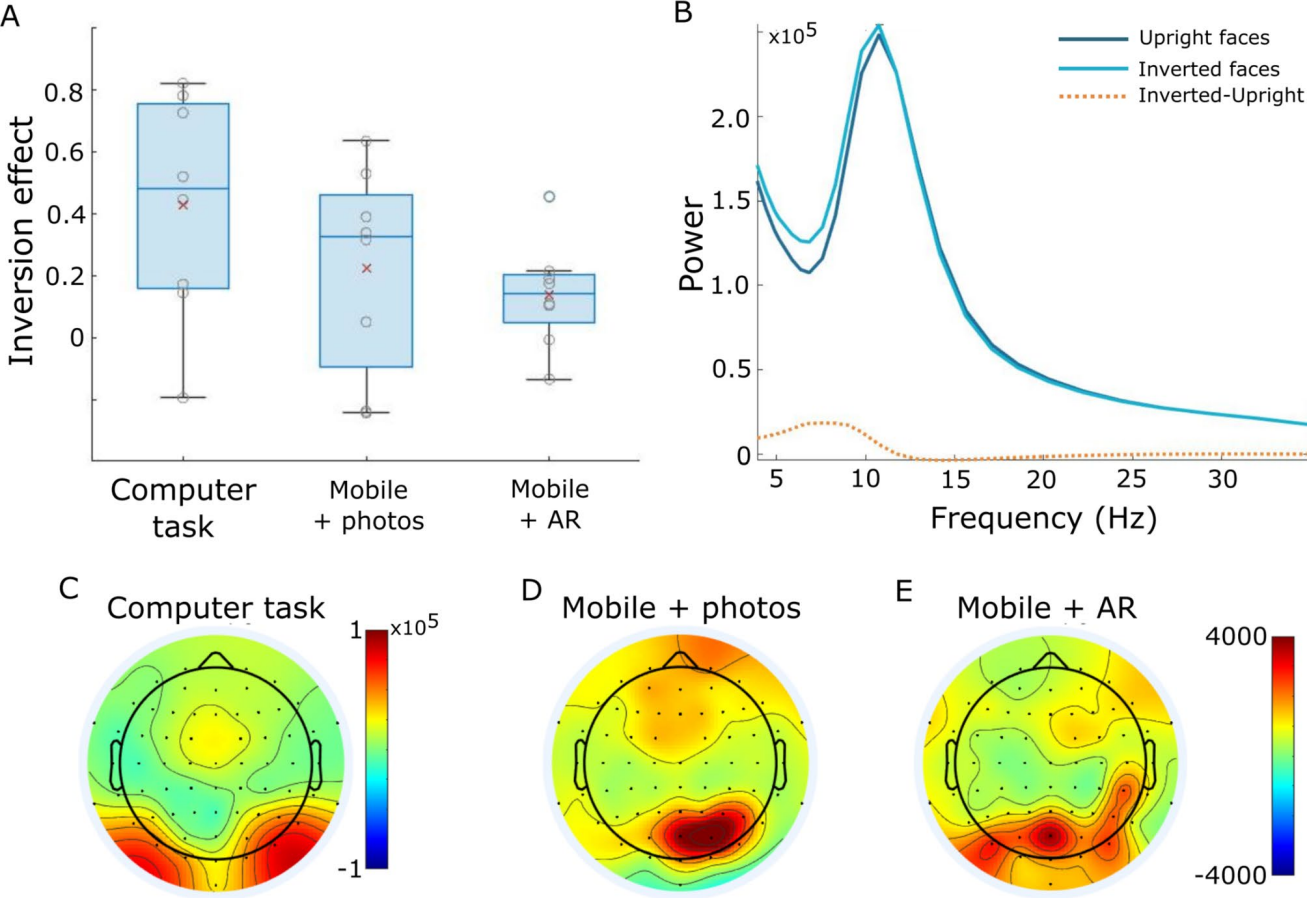
Epoch-based results. (**A**) Face inversion effect sizes for each experimental task based on the posterior electrodes. Red x indicates group mean inversion effect with individual subjects shown by grey circles. (**B**) Spectrogram showing group mean power between 4 and 35 Hz for upright and inverted conditions, and the difference between them. (**C**–**E**) Topographies showing mean power difference for inverted-upright faces between 5 and 15 Hz for the computer task (**C**), mEEG + photos (**D**) and mEEG + AR (**E**).

Our initial analyses looked to replicate the well-characterised face inversion effect in a standard laboratory setting. We calculated low frequency power for upright and inverted face trials for the first 400 ms of the face appearing, averaged the power across 5 to 15 Hz, and averaged these values across a set of posterior electrodes. To test for changes in power for upright and inverted faces, linear mixed effects modelling was used, showing that EEG low frequency power over posterior electrodes was significantly greater for inverted faces compared to upright faces (mean difference = 0.404, t(1427) = 8.27, *p* < 0.0001; Fig. 3A). This result is expected, and replicates previous reports^26,27^. Plotting EEG power across frequencies further indicated that these increases in low frequency power peaked near 10 Hz, and differences between inverted and upright faces were principally between 5 and 12 Hz (Fig. 3B). Finally, to examine face inversion effects beyond the posterior electrodes, inversion effects were calculated for each electrode, showing greater power for inverted compared to upright faces that were primarily located at posterior central and posterior lateral electrodes (Fig. 3C).

#### Task 2: mEEG + photos

Our next analysis asked whether face inversion effects could be seen in a more naturalistic setting using mobile EEG while participants viewed upright and inverted pictures of faces placed on the walls. Black and white photos of faces were placed on the wall at various locations along a corridor and in the adjoining rooms. Participants were fitted with the mobile EEG system and a head-mounted camera, and repeatedly walked along the corridor viewing the faces, pushing a button when they were looking at each face image. We used these button presses to segment the continuous EEG recording into 4 s epochs centred on the button press, and extracted the mean low frequency power for each face event.

Following the analysis used for the computer-based task, we first performed a linear mixed effects analysis of EEG power averaged across posterior electrodes. This revealed a significant effect of face inversion, with greater power for inverted faces compared to upright faces (mean difference = 0.190, t(597) = 2.38, *p* = 0.017; Fig. 3A). Across the scalp, inversion effects were greatest over posterior central electrodes and frontal electrodes (Fig. 3D). Effect sizes were maximum over posterior electrodes, and were approximately half the size of the effect sizes seen during the computer-based task (Fig. 3A). This shows that face inversion effects are detectable using mobile EEG in a natural indoor setting.

#### Task 3: mEEG + AR

An important issue for studies using mobile EEG in natural settings, is the ability to manipulate the environment for the purposes of the experiment. Here, we combine mobile EEG with a head-mounted AR system which enables us to present virtual objects embedded within the real environment. Using the same corridor setting as in task 2, upright and inverted virtual heads were placed at various locations along the corridor and in the adjoining rooms. Like in task 2, participants repeatedly viewed the faces and pressed a button when they were fixating on the face. Again, the continuous data were segmented using the timings from the button presses, creating 4 s epochs centred on the button press, before calculating low frequency power for each face event.

A linear mixed effects model of EEG power averaged over posterior electrodes showed a significant effect of face inversion, with greater power for inverted compared to upright faces (mean difference = 0.170, t(828) = 2.54, *p* = 0.0112; Fig. 3A). Across the scalp, the inversion effect was maximal over posterior central electrodes (Fig. 3E). These effects were partially overlapping with those seen in the computer-based and Mobile + photos tasks, with similar effect sizes in both mobile tasks. Through the combination of mobile EEG and head-mounted AR, this analysis establishes a feasible approach to studying cognitive processes in natural, real environments in which the participant is immersed.

### GLM-based analysis

While the epoch-based analyses establish that mobile EEG and AR are a feasible combination for cognitive neuroscience, an important step is being able to relate the changing visual world to dynamic neural signals in a more flexible manner. This can be achieved through relating the time-varying EEG signals to a time-varying signature reflecting cognitive events. As such, we employed an approach to link face inversion to the EEG signals reminiscent of research relating BOLD responses to visual events using a GLM approach^19,20^.

**Figure 4:**
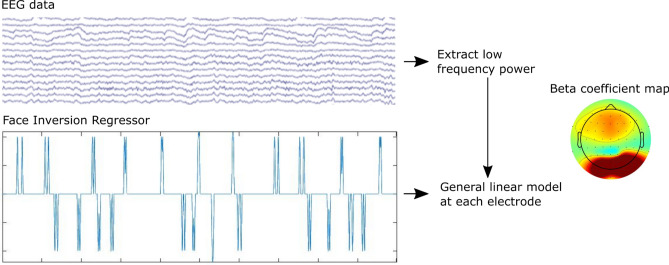
GLM-based approach. The preprocessed, continuous EEG signals were converted to low frequency power over time. A face inversion channel, the same length as the EEG data, was created where a positive spike occurred when an inverted face was seen, and a negative spike occurred when an upright face was seen. This channel was convolved with a 2 s hamming window to create the face inversion regressor. The low frequency EEG signals were modelled by the face regressor using a general linear model at each electrode, resulting in a beta coefficient map for each participant.

**Figure 5:**
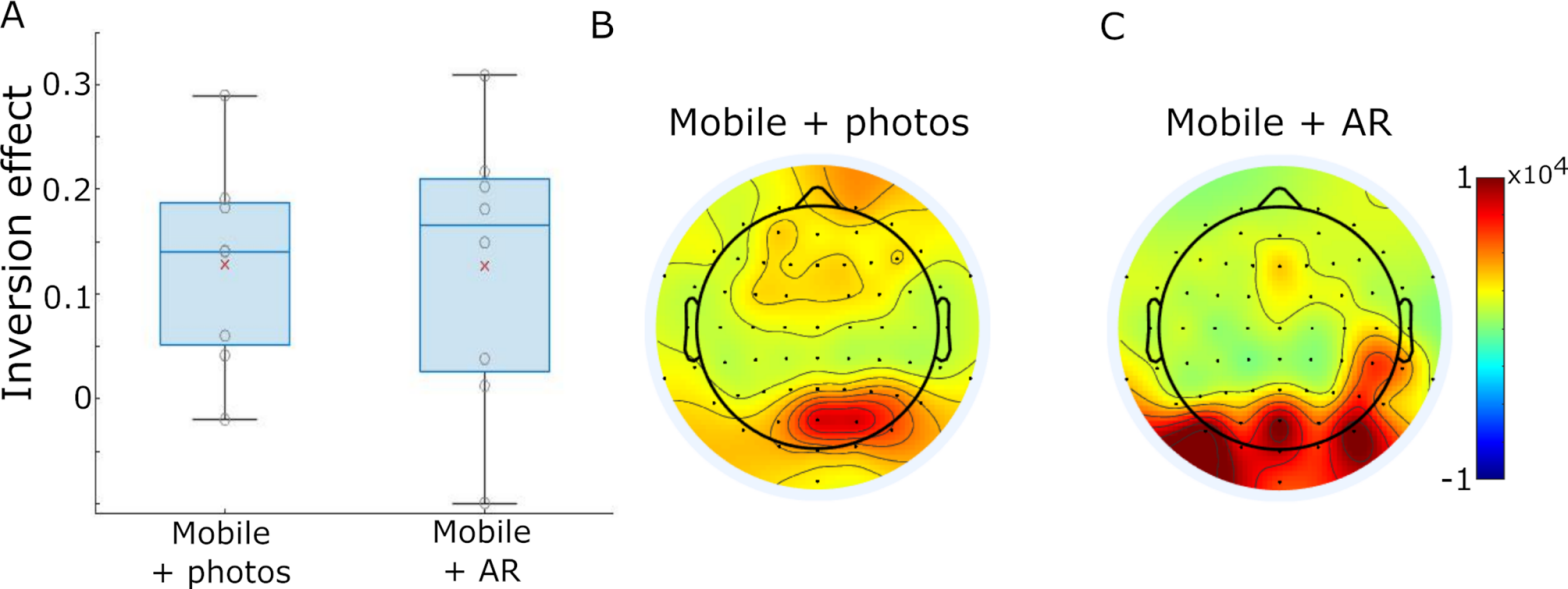
GLM-based results. (**A**) Face inversion effect sizes for the mobile experimental tasks based on the posterior electrodes. Red x indicates group mean inversion effect with individual subjects shown by grey circles. (**B**) Topography showing mean power difference for inverted-upright faces between 5 and 15 Hz for the mEEG + photos task, and (**C**) the mEEG + AR task.

GLMs including a face inversion regressor were run for each participant (Fig. 4). For the Mobile + photos task, EEG low frequency power was averaged across posterior sensors prior to the GLM. This revealed a significant effect of face inversion, with greater power for inverted faces compared to upright faces (mean difference = 0.128, t(7) = 3.71, *p* = 0.0076; Fig. 5A). In addition, effect sizes were calculated at each electrode which showed greater power for inverted compared to upright faces over posterior electrodes (Fig. 5B). For the Mobile + AR task, the GLM based on posterior sensors also revealed a significant effect of face inversion, with greater power for inverted faces compared to upright faces (mean difference = 0.126, t(7) = 2.7, *p* = 0.031; Fig. 5A). Additionally, face inversion effects were greatest over posterior electrodes (Fig. 5C). It is also notable that the effect size maps across electrodes are highly similar for the epoch-based and GLM-based analyses.

In summary, we show that combining continuous event markers of perception, with continuous mobile EEG can reveal face inversion effects similar to those obtained using an epoch-based approach and contrasting conditions. This provides an approach that can be very flexible whereby multiple events and event types could be simultaneously modelled during mobile EEG.

### Participant motion and EEG

**Figure 6:**
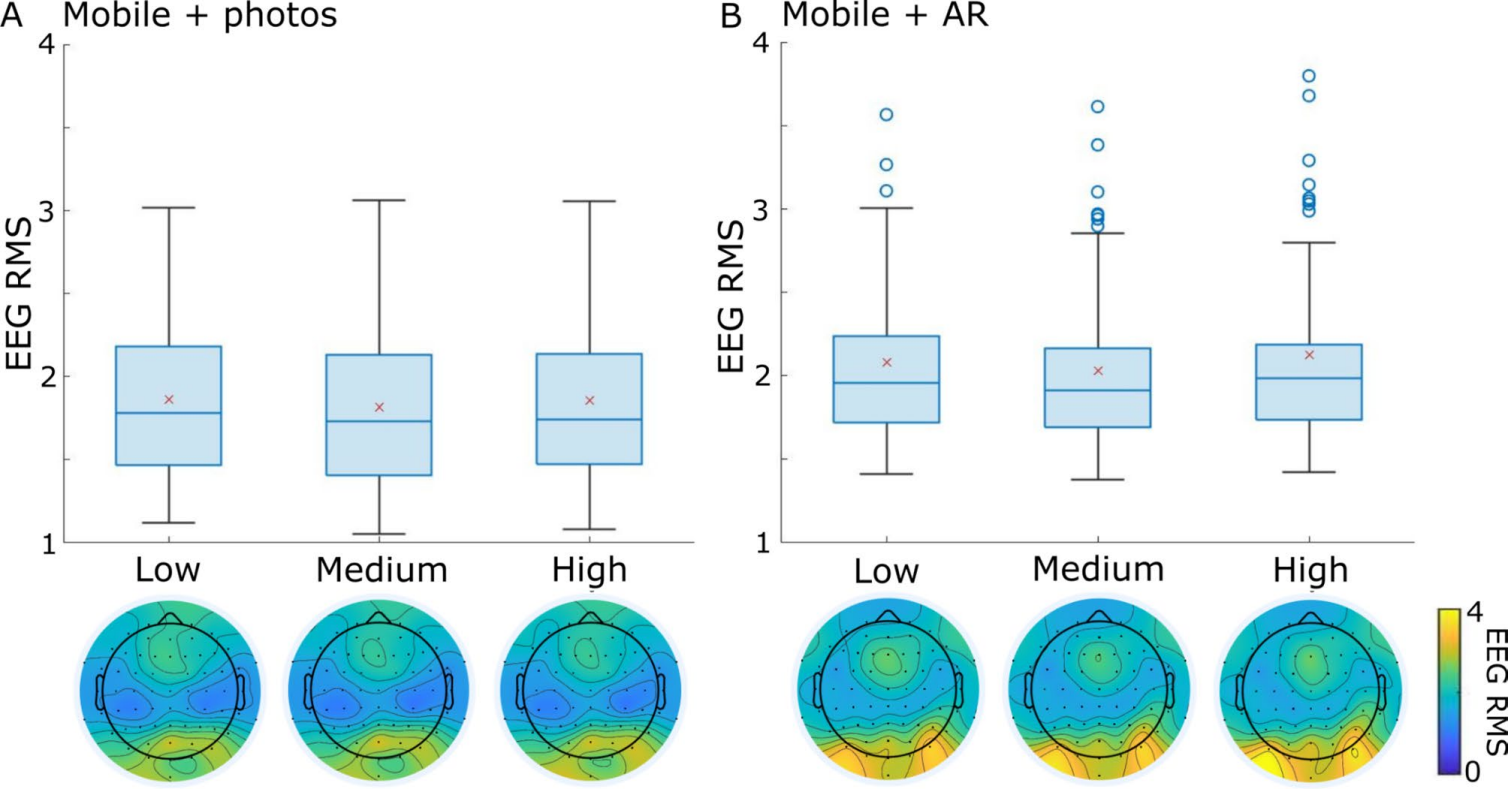
EEG RMS during low, medium and high participant motion for (**A**) the Mobile + photos task and (**B**) the Mobile + AR task. Red x indicates mean RMS over electrodes and participants with boxplots showing the distribution of RMS values across electrodes. Blue circles show outlier electrodes based on RMS values (not motion). Topographies show electrode RMS values for each motion RMS bin.

As a final assessment of the EEG signals during mobile recording, we assessed whether information from the EEG systems accelerometer channels related to signals from the EEG electrodes. To do this, we split the continuous data into 2 s non-overlapping segments, and calculated the root mean square (RMS) for the accelerometers (motion RMS) and each EEG electrode. Motion RMS was divided into low, medium and high groups, and related to EEG RMS using a linear mixed effects model. EEG RMS signals appear stable over the 3 motion levels for both mobile tasks (Fig. 6A,B), with no significant effects of motion level on EEG RMS (Mobile + photos: estimate − 0.003, t(1534) = − 0.12, *p* = 0.90; Mobile + AR: estimate 0.022, t(1534) = 0.79, *p* = 0.43). Note, equivalent results are present using a measure of signal variance rather than RMS.

## Discussion

In the current study, we combined mobile EEG and head-mounted AR to establish a feasible approach to studying cognitive processes in natural, real environments in which the participant is immersed. In order to establish the feasibility of our approach, participants completed three EEG face inversion tasks: (1) a computer-based task, (2) a mobile task with photographs of faces on the walls, and (3) a mobile task where virtual faces were presented through the head-mounted AR device. In all tasks, and in both an epoch-based analysis and a GLM-based analysis that uses continuous EEG data, we see increased low-frequency power over posterior electrodes for inverted faces compared to upright faces, replicating known face inversion effects^26,27^ but in a novel experimental paradigm. Importantly, our analyses clearly show face inversion effects can be identified during free moving EEG paradigms. Our research shows that combining whole-head mobile EEG and head-mounted AR is a feasible approach to studying cognitive processes in natural and dynamic environments, which could help open the door to studying a variety of cognitive factors in real environments, whilst also allowing for the control of visual aspects of those environments using AR.

In order to successfully analyse perception during mobile EEG, we departed from typical EEG analyses. In most EEG paradigms there is an event with a definite onset, for example when a picture might appear on screen having previously not been present. This would be akin to most lab-based studies of perception, and our data from task 1. However, for our mobile EEG tasks (2 and 3), items did not appear and disappear but remained in the environment and could be seen as soon as they appeared in the participants’ field of view. As a consequence, items could be seen from a distance and approached, and perceived for extended periods or only fleetingly. This means we did not have an ‘event onset’ as such, and the concept of an evoked response did not clearly apply. Arguably, this more closely mirrors natural perception and behaviour, but presents challenges for data analysis. To overcome this issue, participants pushed a button when they arrived at, and were looking at the stimuli. Using this marker for the epoch-based analysis, we extracted oscillatory power for each trial over a 2 s period to test for power changes between our conditions without requiring a time-locked point. However, we will also likely miss numerous times when each item was fixated upon, and when the item was first seen. An alternative, more flexible approach, could involve the use of eye-tracking to define the time-point when a stimulus is fixated upon, with this information being used to construct fixation-related potentials^28,29^. As such, future studies could look to incorporate eye-tracking measures during mobile EEG and AR for more precise object recognition effects. In addition to eye-tracking, monitoring participant head direction and movement could be included as further measures to incorporate into data analysis. While we found no relationship between motion and EEG signals, we used a general measure of motion as the accelerometers were positioned on the back, not head. Other studies have used head mounted accelerometers, seeing no relationship between head motion and cognition (e.g.^13^), which could be incorporated in future studies.

While this approach was successful for our epoch-based analysis, alternative approaches are needed to take advantage of the continuous nature of perception, where multiple different events occur distributed over time. In our GLM-based analysis, we demonstrate an approach to relate time-varying neural signals to time-varying measures of perception, again based on face inversion. Using the continuous processed EEG signals, rather than discrete epochs, we were successful in relating the time-varying perceptual characteristics of the visual environment to the time-varying neural characteristics measured with EEG. We achieved this using an GLM to relate EEG signals to a continuous measure capturing face inversion, with additional regressors for motion parameters of no interest. With this analysis we replicated the face inversion findings of the epoch-based approach, where the effect sizes were comparable across the analyses as were the topographic distributions of effect sizes. Although this was a simplified example, the GLM approach appears effective for studying relationships between the visual world and neural signals, allowing greater flexibility to relate continuous perpetual events to continuous EEG signals. This approach followed similar applications in fMRI (e.g.^20^) and MEG (e.g.^21^) but in this case expanded the application to mobile, freely moving settings.

In this study, we only examined within-condition effects, although our results might suggest that effect sizes in the mobile conditions are reduced compared to a laboratory setting. However, many factors could have contributed to this, such as different numbers of trials, different stimuli and the different data recording modes (mobile vs. stationary). Despite this, we observe face inversion effects of similar morphology across the different conditions regardless of these differing paradigms, allowing us to argue that we are recording a common face inversion mechanism across all conditions.

The use of AR in mobile EEG experiments designed to study perception and cognition is a novel approach. Unlike VR, where both objects and the environment are computer generated, AR allows for virtual objects to be embedded into complex real-world environments, opening up new possibilities of controlled experimental designs in natural settings. For example, it allows for the navigation of personally familiar routes taken on a frequent basis whilst changing the items seen on the route or the path taken. One question that becomes relevant when considering AR (or VR), is whether virtual objects elicit neural responses similar to seeing real objects. A similar question has been raised concerning neuroimaging studies based on 2D images of objects compared to perceiving real 3D objects, with sometimes different effects across the conditions^30^. In the current study we used 3D virtual faces that resemble human faces, but did not have all the characteristics of natural faces. Yet our results demonstrate similar neural responses to virtual faces and to images of faces, demonstrating that AR is a feasible option for presenting 3D stimuli that can be reliably studied with EEG.

Whilst our study could have been conducted without AR, we believe that the combination of AR and mEEG provides a powerful paradigm for psychology and cognitive neuroscience. With increasingly complex tasks and environments, it becomes more important to retain some experimental control to both provide participants with similar experiences and enable the researchers to modify perception in a flexible manner. The increasing use of AR for experiments could allow more research to be conducted outside the lab, where participants retain a sense of agency and immersion in the environment. Current research suggests AR is more engaging than VR, with potential improvements in memory encoding^17^, and is sensitive to changes in attentional states^31^. In addition, head-mounted AR is immersive yet keeps the participate in the real world, and compared to VR, is more comfortable for longer sessions and is less likely to induce nausea. Combining mEEG and AR is especially suited to investigate cognitive processes that can be studied by placing and manipulating objects in a real-world setting, and might benefit treatments of, for example, anxiety conditions that are linked to objects appearing in specific locations. On the other hand, for questions that require the manipulation of environments as opposed to objects, virtual reality would be better suited than AR, as it allows to create and directly manipulate whole scenes. We expect that future research will achieve a better understanding of cognitive processes by combining traditional approaches with emerging technologies like AR and VR. One important avenue of future research will be the bilateral translation between lab-based and real-world studies using EEG and AR. For the *lab-to-life* direction, experimenters could utilise the stimuli and trial screens used in the lab, and present them as virtual stimuli to be seen through head-mounted displays. Such an approach would allow for the evaluation of whether lab-based effects are also seen in real-world settings. We can also think about translation in the opposite direction. Paradigms presented in AR allow for mixed reality video to be recorded with the virtual stimuli in the real-background, perceived as they would be in AR. Whilst capturing the perceptual experience of the participants, they also afford the creation of stimuli seen as it would be in AR, but without the participant actively being in the environment. This facilitates the *life-to-lab* translation, in terms of perceptual experience, and allows for real-world effects to be interrogated in a controlled setting, and divergences between the two conditions to be explored.

If research in psychology and cognitive neuroscience is to increasingly adopt AR and EEG approaches, then there are a number of opportunities and pitfalls to consider, a few of which we comment on here. (1) While the approach we have taken here is relatively straightforward, typical paradigms and tasks are more complex. To translate such tasks to immersive devices such as AR requires software suited for this purpose. While software that supports either VR or 3D games can be used and adapted^17,31^ there is the ongoing development of software to specifically allow the creation of psychology experiments for VR and AR purposes^32^, making it more accessible to translate typical paradigms into AR. (2) As mentioned, a further consideration in AR research is whether the device creates an immersive experience, and whether this is important to the research. Here, to enhance ecological validity, we use a head-mounted immersive display, the Hololens 2, where the virtual objects are seen placed in the real-world though lenses close to the eyes. This is in contrast to AR displays on a tablet or smartphone where the participant’s view of the mixed reality depends on how the device is held or positioned. While immersive headsets also allow for more realistic human-object interactions and manipulations, AR through a tablet is more accessible and inexpensive. (3) The integration of AR devices and imaging techniques like EEG requires communication between the devices, and additional peripheral equipment like button boxes or sensors. While in our study we did not require precise time stamps for the appearance of virtual objects, future studies that require synchronised timing between EEG and AR devices will require customised setups to achieve this. These are likely to be hardware specific, depending on the communication ports and modes of the device. (4) Another practical issue with some instances of AR, such as the head-mounted AR display on the Hololens, is that objects can appear with different contrast and clarity depending on the lighting conditions. This can mean an item presented in a sunny outdoor environment might appear different to the item in a dull indoor setup. As a consequence, comparisons between the same item in different environments might be problematic due to the changes in physical appearance. However, smart-phone and tablet AR (i.e. non-immersive) would not suffer from this as the item is rendered on a screen rather than virtually projected into the background as seen by the participant. As a community of researchers begin to utilise AR with EEG, we expect these, and many other issues, will be resolved.

In conclusion, here we show that cognitively relevant neural signals can be detected in AR and mobile EEG paradigms. Similar to lab-based effects, we showed inverted AR faces elicit greater low frequency power compared to upright AR faces while participants freely moved through an indoor office space. The combination of AR and mobile EEG could offer a new paradigm for cognitive neuroscience, whereby cognition can be studied while participants are immersed in natural environments yet experimenters can retain some control over what items people see and how.
